# Effects of Fluoride and 8:2 FTOH on β-Cell Calcium Signaling and Insulin Homeostasis: An Exploratory Study

**DOI:** 10.3390/metabo16070470

**Published:** 2026-07-04

**Authors:** Juliana Sanches Trevizol, Motoki Okamoto, Shohei Yamashita, Nanako Kuriki, Susanne Brueckner, Satoru Shindo, Toshihisa Kawai, Raissa Estefane Vaz Damião, Aline Dionizio, Marilia Afonso Rabelo Buzalaf, Maiko Suzuki

**Affiliations:** 1 Department of Oral Science and Translational Research, College of Dental Medicine, Nova Southeastern University, Fort Lauderdale, FL 33314, USA; julianatrevizol@usp.br (J.S.T.); okamoto.motoki.dent@osaka-u.ac.jp (M.O.); syamashi@nova.edu (S.Y.); kuriki.nanako.dent@osaka-u.ac.jp (N.K.); sbrueckn@nova.edu (S.B.); sshindo1@nova.edu (S.S.); tkawai@nova.edu (T.K.); 2 Department of Biological Sciences, Bauru School of Dentistry, University of São Paulo, Bauru 17012-902, São Paulo, Brazil; restefane901@gmail.com (R.E.V.D.); alinesdionizio@usp.br (A.D.); 3 Department of Restorative Dentistry and Endodontology, Graduate School of Dentistry, Osaka University, Osaka 565-0871, Japan

**Keywords:** fluoride, FTOH, PFAS, β-cells, pancreas, calcium

## Abstract

**Highlights:**

**What are the main findings?**

- Exposure to 8:2 FTOH modulated intracellular Ca^2+^ homeostasis in pancreatic β-cells in a dose-dependent biphasic manner, similarly to fluoride (F), with higher concentrations impairing Ca^2+^ signaling and reducing GPR40 protein expression.
- In vivo pancreatic proteomics revealed alterations in proteins and interaction networks associated with lipid metabolism, oxidative stress, endoplasmic reticulum stress, Ca^2+^ signaling, and insulin-related pathways after 8:2 FTOH exposure.

**What are the implications of the main findings?**

- PFAS metabolites and fluoride could affect pancreatic β-cell function and insulin secretion through complex non-transcriptional mechanisms involving Ca^2+^ regulation and metabolic signaling pathways.
- Low-dose exposures produced limited biological alterations, supporting the concept of hormetic responses and reinforcing the safety of controlled fluoride exposure at physiologically relevant concentrations.

**Abstract:**

**Background/Objectives**: Fluoride (F) is widely used in public water fluoridation to prevent dental caries, and an optimal level of F has been linked to improved glucose metabolism in animal models. Per- and polyfluoroalkyl substances (PFAS), including fluorotelomer alcohols (FTOHs), are persistent environmental contaminants with potential effects on pancreatic function. **Methods**: This in vitro and in vivo study investigated the effects of 8:2 FTOH and F (NaF) on pancreatic β-cells, focusing on Ca^2+^ homeostasis, insulin secretion, and the GPR40 pathway. **Results**: Results showed that 8:2 FTOH alters Ca^2+^ influx in a dose-dependent, biphasic manner, enhancing it at low doses and inhibiting it at high doses, while F increased Ca^2+^ signaling at high doses. High-dose 8:2 FTOH also downregulated GPR40 protein in βTC-6 pancreatic cells and modulated pathways related to lipid metabolism, endoplasmic reticulum stress, and insulin regulation in the mouse pancreas by proteomic analyses (in vivo). **Conclusions**: These findings exploratory indicate that both PFAS and F can impact β-cell function through complex mechanisms, potentially affecting Ca^2+^ homeostasis. This work highlights the hormesis effect of F and provides novel insights into the pancreatic effects of environmentally relevant PFAS exposures, emphasizing the need for further mechanistic studies at low, human-relevant doses.

## 1. Introduction

Fluorotelomer alcohols (FTOHs) are fluorinated telomer-based chemicals widely used in industry to produce polymers and surfactants due to their high chemical stability conferred by strong C-F bonds [1,2,3,4]. During degradation, 8:2 FTOHs are metabolized to perfluorooctanoic acid (PFOA) and F. PFOA has been consistently detected in human blood and plasma [5,6,7]. 8:2 FTOH is precursor of PFOA, and both belong to the per- and polyfluoroalkyl substances (PFAS), a class of persistent, bioaccumulative, and environmentally widespread contaminants with growing public health concern [4].

FTOH exposure has been associated with hepatotoxicity, increased proliferation of cancer cells and altered estrogenic activity [8,9,10]. Epidemiological studies link PFAS exposure to increased cholesterol levels, liver injury, chronic kidney disease, and glucose intolerance. Insulin secretion induced by Perfluorooctane Sulfonate (PFOS), another PFAS, is mediated through activation of G protein-coupled receptor 40 (GPR40) [11,12,13]. Experimental evidence shows that PFOS can stimulate insulin synthesis in pancreatic β-cells through G protein signaling, activation of PIP2, IP3, and DAG, opening of Ca^2+^ channels in the endoplasmic reticulum, and PKC activation. However, findings regarding PFOA and glucose homeostasis in vivo remain inconsistent [13], with studies reporting both increased fasting glucose and improved glucose tolerance and insulin sensitivity, depending on dose and exposure conditions [14,15]. Mice exposed to high physiologically relevant doses of PFOA had increased fasting glucose levels, mild glucose intolerance, and reduced insulin sensitivity [16]. Therefore, high doses of PFOA consistently cause fasting hyperglycemia, but more studies are needed to resolve the variability in glucose tolerance and insulin secretion results.

Interestingly toxicological effects of FTOHs may stem not from the parent compounds but also from their metabolites. Notably, no studies have to date evaluated their direct impact on pancreatic cells. This is particularly relevant because 8:2 FTOH can release fluoride ions during degradation to PFAS [17].

Fluoride is widely used in artificial water fluoridation due to its proven effectiveness and safety in preventing dental caries [18,19]. Beyond its dental benefits, F influences systemic metabolism. Proteomic analyses of pancreatic islets from NOD mice exposed to water containing 10 mg F/L for 14 weeks identified unique expression patterns in metabolic proteins, including acetyl-CoA carboxylase 2 (ACC2), which is involved in lipid metabolism and regulation of histone acetylation. Fluoride exposure markedly increased histone acetylation and levels of histone acetyltransferases KAT6B and KAT8, suggesting a potential epigenetic role in pancreatic tissue [20].

Additional findings from our group showed reduced blood glucose in female NOD mice treated with water containing 10 mg F/L for 14 weeks. This was accompanied by the absence of Phosphoenolpyruvate carboxykinase (PEPCK) expression in the liver. As F inhibits enolase, a key glycolytic enzyme, the observed reduction in glucose levels may reflect downstream suppression of PEPCK [21]. Studies in male NOD mice treated with water containing 0, 10 or 50 mg F/L for 21 days revealed dose-dependent improvements in glucose metabolism. Animals receiving water containing 10 mg F/L (a concentration equivalent to artificially fluoridated water for humans [22,23]) had significantly lower blood glucose and a significantly higher % of β-cell function compared to controls, in addition to significantly altered expression of proteins in the liver and gastrocnemius muscle of animals, with a notable presence of Glutathione S-transferase P2 and Heat shock-related 70 kDa protein 2, involved in antioxidant defense in the livers of animals treated with 10 mgF/L [21,22,24]. Similarly, low-dose F enhanced insulin sensitivity in streptozotocin-induced diabetic rats, supporting a broader metabolic effect [25].

Given the metabolic relevance of both PFAS and F, and the absence of studies addressing FTOH effects in pancreatic tissue, this study investigates in vivo the mechanisms by which 8:2 FTOH affects the pancreas through proteomic analysis, as well as the effects of F at physiologically relevant concentrations on GPR40 and peroxisome proliferator-activated receptor gamma (PPARγ) in βTC-6 pancreatic cells. PPARγ is a known regulator of β-cell gene expression and glucose metabolism [13].

## 2. Materials and Methods

### 2.1. Animals and Treatment

Five-week-old male C57BL/6J mice were obtained following protocol approval by the Institutional Animal Care and Use Committee (IACUC) of Nova Southeastern University (Protocol No. 2023.02.MSuz1), certified by the Association for Assessment and Accreditation of Laboratory Animal Care International (AAALAC), and were obtained from The Jackson Laboratory (Bar Harbor, ME, USA) following ARRIVE 2.0 guidelines. The male mice were randomly assigned to 3 groups (*n* = 6/group)—control (0 mg/kg/day), low dose (50 mg/kg/day), or high dose (125 mg/kg/day) of 8:2 FTOH administered via oral gavage for 90 days—based on a previously established protocol and studies conducted by our research group [26,27,28]. 8:2 FTOH (Cat. H084525G, Tokyo Chemical Industry, Tokyo, Japan), chemically identified as 1H,1H,2H,2H-heptadecafluoro-1-decane, was prepared in a vehicle solution of 0.5% carboxymethylcellulose (Sigma-Aldrich, Saint Luis, MO, USA) [27,28]. All animals were maintained with free access to food and water ad libitum, under a 12:12 h light–dark cycle in an acclimated room with controlled temperature and relative humidity.

All animals were euthanized at 13 weeks of age, and blood was collected by cardiac puncture to obtain plasma, which was stored at −20 °C for insulin analyses using Mouse Ultrasensitive Insulin ELISA-ALPCO^®^ (Catalog #80-INSMSU-E01,E10, Salem, NH, USA). The pancreas was then collected and stored at −80 °C for proteomic analysis.

### 2.2. Proteomic and Bioinformatic Analyses

Microtubes containing pancreas samples with 10% (*v*/*v*) protease inhibitor were prepared for proteomic analysis as previously described [25]. Peptide identification was performed on a nanoAcquity UPLC-Xevo G2 QTof MS system (Waters, Manchester, UK), as previously reported [29,30]. LC-MSE data were processed and analyzed using ProteinLynx Global Server (PLGS) version 3.0.3 software and the updated *Mus musculus* database (UniProtKB). Peptide and protein identifications were filtered using a false discovery rate (FDR) threshold of 2%. Differential protein expression and abundance ratios between experimental groups were determined using the Waters Protein Expression System integrated into PLGS [29], which applies a probabilistic model based on peptide ion intensities to estimate the likelihood of differential expression. Proteins were considered significantly downregulated when *p* < 0.05 and significantly upregulated when 1 − *p* > 0.95. Protein–protein interaction (PPI) networks were constructed using CYTOSCAPE^®^ 3.10.0 version (Java^®^ 8 platform), with functional enrichment and clustering analyses performed through the ClueGO and ClusterMarker^®^ plugins, respectively.

The mass spectrometry proteomics data have been deposited at the ProteomeXchange Consortium via the PRIDE partner repository [31] with the dataset identifier PXD073286.

### 2.3. Cell Culture and Treatment

The mouse pancreatic β cell line βTC-6 was obtained from the American Type Culture Collection (ATCC CRL-3605, Manassas, VA, USA) and maintained in Dulbecco’s Modified Eagle Medium (DMEM, high glucose), supplemented with 15% fetal bovine serum (FBS, Gibco, Thermo Fisher, Grand Island, NY, USA) and 1% penicillin/streptomycin (Gibco), in a 37 °C and 5% CO2 incubator. All assays were performed in biological triplicate.

The following groups were formed: (1) no treatment (negative control); (2) 1 µM F (as NaF); (3) 5 µM F; (4) 10 µM 8:2 FTOH; and (5) 50 µM 8:2 FTOH. The FTOH concentrations used were determined by MTT and LDH viability assays after 24 h of treatment. 8:2 FTOH was dissolved in 0.04% DMSO, which was also used as the vehicle for the control group.

The F concentrations (1 µM and 5 µM) evaluated represent levels expected in human plasma in individuals ingesting water containing 1 and 3 mg F/L, respectively [32]. These concentrations correspond to 5–10 or 25–50 mg F/L administered to rodents, as they metabolize F 5–10 times faster than humans [23]. These concentrations therefore reflect F levels in optimally fluoridated areas and in regions with endemic fluorosis, respectively.

### 2.4. Citotoxicity Analysis—MTT and LDH Assays

Approximately 1.4 × 10^4^ βTC-6 cells were seeded at 96-well plates for 24 h. Cell viability was assessed by MTT (3-(4,5-dimethylthiazol-2-yl)-2,5-diphenyltetrazolium bromide) assay (Sigma-Aldrich^®^, St. Louis, MO, USA). After the pilot assay, 8:2 FTOH was tested at concentrations of 0.001, 0.01, 0.1, 1, 10, 50, 100, 200, 300 and 500 μM for 24 h (time previously optimized). Based on the MTT results, concentrations of 10 and 50 μM were selected for subsequent experiments, corresponding approximately to concentrations that induced low and moderate reductions in cell viability (IC_10_ and IC_50_, respectively). These concentrations were chosen to evaluate cellular responses across a biologically relevant range of toxicity and to investigate potential molecular mechanisms associated with 8:2 FTOH exposure.

The lactate dehydrogenase (LDH) cytotoxicity assay was performed according to the manufacturer’s instructions and the experiments were both repeated three times.

### 2.5. Insulin and Ca^2+^ Intracellular Level Detection Assay

βTC-6 cells were plated at a density of 8 × 10^5^ cells/well in a 12-well plate and cultured for 24 h. The cells were washed with Hank’s Balanced Salt Solution (HBSS; containing 0.1% BSA, pH 7.0) and incubated in HBSS for 30 min to remove residual glucose from the medium. Cells were then exposed to defined concentrations of 8:2 FTOH and NaF in HBSS containing 16.7 mM glucose for 1 h, following an adaptation of the protocol by Zhang, Duan, Sun and Sun [13]. Insulin levels were measured using the Ultra Sensitive Mouse Insulin ELISA Kit (Catalog #90080, Crystal Chem, Itasca, IL, USA), and the cells were lysed for protein normalization.

For intracellular Ca^2+^ level detection, the βTC-6 cells were seeded (1.4 × 10^4^ cells/well) in 96-well black-walled, clear-bottom plates. The cells were loaded with 2 μM Fluo 8-AM^®^ No Wash Calcium Assay Kit (Catalog #21080, AAT Bioquest, Pleasanton, CA, USA), in calcium- and glucose-free HBSS for 1 h at 37 °C, followed by 30 min at room temperature under light protection. Afterward, the cells were treated or not (control group) with 8:2 FTOH or NaF in HBSS containing glucose, and fluorescence intensity was measured with excitation at 490 nm and emission at 525 nm every 10 s using a FilterMax F5 Microplate Reader (Molecular Devices, San Jose, CA, USA), following the manufacturer’s protocol. Ionomycin was used as a positive control [33].

### 2.6. Rt-qPCR Analysis

Rt-qPCR was performed as described previously [31,32]. Briefly, total RNA was extracted using the Direct-zol RNA MiniPrep kit (Zymo Research Corp, Irvine, CA, USA), and cDNA was synthesized using the iScript cDNA Synthesis Kit (Bio-Rad Laboratories Inc., Hercules, CA, USA) with a C1000 Touch thermal cycler (Bio-Rad). cDNA was then subjected to qPCR amplification using a QuantStudio 3 (Thermo Fisher Scientific, Grand Island, NY, USA). Primer sequences are provided in Supplementary Table S1. Cycle threshold (Ct) values were obtained, and data were normalized to glyceraldehyde-3-phosphate dehydrogenase (GAPDH) expression using the 2^−ΔΔCT^ method to calculate relative mRNA levels [13,34,35]. Four to five independent experiments were conducted.

The selection of an appropriate reference gene for RT-qPCR normalization was carefully assessed to minimize potential bias associated with treatment-induced transcriptional variability. Candidate housekeeping genes (GAPDH, 18S, and B2M) were evaluated using triplicate measurements, and their expression stability was analyzed with RefFinder, which integrates the geNorm, NormFinder, BestKeeper, and comparative ΔCt algorithms [14]. GAPDH showed the highest overall stability ranking among the genes evaluated and was therefore chosen as the endogenous reference gene for normalization of mRNA expression levels throughout the study.

### 2.7. Western Blotting

βTC-6 cells (1 × 10^6^ cells/dish) were plated in 10 cm dishes and cultured for 24 h. Cells were then lysed, and proteins were extracted using RIPA buffer containing 1% HaltTM protease inhibitor (Thermo Fisher Scientific, Grand Island, NY, USA), followed by quantification using the BCA protein assay kit. Protein samples were subjected to Western blot analysis as reported previously [34,35].

The following primary antibodies were used: rabbit anti-GPR40 (1:200, Catalog #PAS-115321, Thermo Fisher Scientific), rabbit anti-PPARγ (1:500, Catalog #16643-1-AP, Proteintech, Rosemont, IL, USA) and rabbit anti-β-actin (1:1000, Catalog #4970S, Cell Signaling Technology, Boston, MA, USA). The secondary antibody used was HRP-conjugated anti-rabbit IgG (1:2000, Catalog #7074, Cell Signaling Technology). Chemiluminescence signal detection was performed using the Azure 400 Fluorescent Western Blot Imager (Azure Systems, Dublin, CA, USA). Band densities were quantified using ImageJ.JS (ImageJ 1.53m version, National Institutes of Health, Bethesda, MD, USA). Relative protein expression data are presented as mean ± SD. Precision Plus Protein^TM^ All Blue Prestained Protein Standard (Catalog #1610373, Bio-Rad) was used. Three independent assays were performed for each experiment.

### 2.8. Statistical Analysis

After checking for normality and homogeneity of variances, data were analyzed using one-way ANOVA and Tukey’s post hoc multiple comparison test (GraphPad Prism 8.0.2). Statistical significance was defined as *p* < 0.05.

## 3. Results

### 3.1. Cell Viability Assessment by MTT and LDH Assays

**Figure 1:**
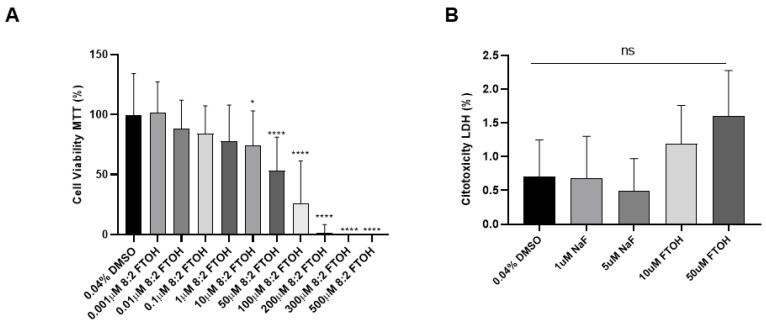
Determination of the cytotoxic effect and IC_50_ of 8:2 FTOH in βTC-6 cells. (**A**) Cell viability was evaluated by the MTT reduction assay after 24 h of exposure to increasing concentrations of 8:2 FTOH (control—0.04% DMSO, 0.001 µM, 0.01 µM, 0.1 µM, 1 µM, 10 µM, 50 µM, 100 µM, 200 µM, 300 µM and 500 µM 8:2 FTOH). The MTT assay was used to determine the half-maximal inhibitory concentration (IC_50_) of 8:2 FTOH. (**B**) Cytotoxicity was assessed by measuring lactate dehydrogenase (LDH) release after 24 h of exposure to the indicated concentrations of 8:2 FTOH (10 µM and 50 µM) and NaF (1 µM and 5 µM F). Data are presented as mean ± standard deviation (SD) from 18 independent observations (*n* = 18). Statistical comparisons were performed against the vehicle control group (0.04% DMSO). Significant differences are indicated as * *p* < 0.05 and **** *p* < 0.0001; ns, not statistically significant.

The cytotoxicity effect of 8:2 FTOH was first tested at concentrations of 0.001, 0.01, 0.1, 1, 10, 50, 100, 200, 300 and 500 μM and the half-maximal inhibitory concentration (IC50) was identified at 224.7 μM. After cytotoxicity analysis, doses of 10 and 50 μM of 8:2 FTOH were chosen. The results (%) of MTT cell viability were expressed as mean ± SD and showed significant reductions at 10 μM (*p* < 0.0162) and at 50, 100, 200, 300 and 500 μM (*p* < 0.0001) (Figure 1A) when compared with the control group.

Furthermore, in the LDH assay, no statistically significant differences were observed in % cell viability between the 8:2 FTOH or NaF (F) treatment groups and the control group (Figure 1B). This was expected, as the F doses (1 and 5 μM) simulated the F concentrations found in plasma of humans ingesting water containing 1 and 5 mg F/L, which are found in areas of optimal artificial fluoridation and in endemic regions of fluorosis, respectively. The chosen 8:2 FTOH concentrations (10 and 50 μM) also reflect low and high exposure scenarios.

### 3.2. Insulin Secretion and Ca^2+^ Intracellular in βTC-6 Cells and In Vivo

**Figure 2:**
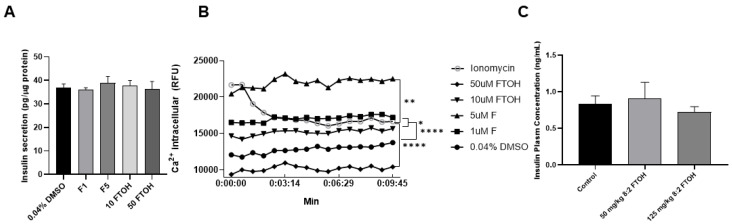
Insulin concentration and Ca^2+^ intracellular levels. (**A**) Insulin secretion normalized with total protein in βTC-cells. (**B**) [Ca^2+^]_i_ dynamic with 16.7 mM of glucose in βTC cells treated or not (control group), with different doses of 8:2 FTOH and NaF in a period of 24 h. (**C**) Insulin plasma concentration administered (50 mg/kg and 125 mg/kg 8:2 FTOH) or not (control) in animals over a period of 90 days *(n* = 3). Values are expressed as mean ± SD for (**A**,**C**) and only mean SEM SD for (**B**). * *p* < 0.05, ** *p* < 0.01 and **** *p* < 0.0001 represent statistical differences when compared to control for (**A**,**C**), or positive control (ionomycin) in (**B**).

Insulin secretion in βTC-6 cells was normalized to total protein content and expressed as mean ± SD. No statistically significant differences were observed between treated groups (1 µM and 5 µM F; 10 µM and 50 µM 8:2 FTOH and the control group (0.04% DMSO)) after 24 h of treatment (*p* = 0.5012; F = 0.8964) (Figure 2A).

Intracellular Ca^2+^ concentration ([Ca^2+^]_i_) was analyzed using two-way ANOVA with TIME and TREATMENT as factors, followed by Dunnett’s post hoc multiple comparison test, and values are expressed as mean (Figure 2B). Significant differences were observed for 5 µM F (*p* = 0.0053), 10 µM 8:2 FTOH (*p* = 0.0228), and 50 µM 8:2 FTOH (*p* < 0.0001) compared with the positive control ionomycin at the 10 min time point. No significant difference was found for the 1 µM F group in βTC-6 cells (*p* = 0.8167). The 5 µM F group showed the highest [Ca^2+^]_i_ (21.895 ± 702).

Regarding plasma insulin concentration in animals treated for 90 days with low (50 mg/kg) or high (125 mg/kg) doses of 8:2 FTOH, no statistically significant differences were observed compared with the control group (Figure 2C, *p* = 0.1691; F = 2.023).

### 3.3. Western Blotting and Real-Time qPCR Analysis of GPR40 and PPARγ Expression in βTC-6 Cells

**Figure 3:**
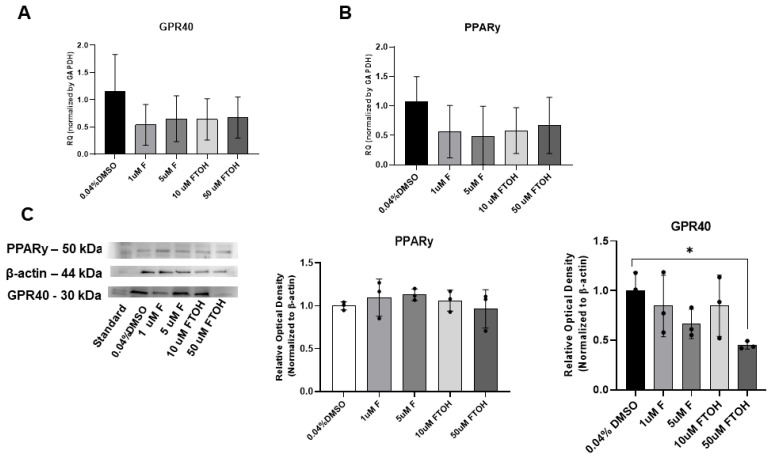
Expression of GPR-40 and PPARγ in βTC-6 cells treated or not (control group) with different concentrations of NaF or 8:2 FTOH for a period of 24 h. (**A**) mRNA levels of GPR40 gene expression by RT-PCR and (**B**) mRNA levels of PPARγ gene expression by RT-PCR, with GAPDH as the reference gene for normalized relative quantification. (**C**) GPR40 (30 kDa) and PPARγ (50 kDa) levels were detected by Western blot. Relative protein levels were normalized by β-actin (44 kDa). Values are expressed as mean ± SD. * *p* < 0.05 represents a statistically significant difference compared to the control group. *n* = 5 for RT-PCR or *n* = 3 for Western blot.

The effects of F or 8:2 FTOH (various doses) on GPR40 (Figure 3A) and PPARγ (Figure 3B) transcript levels after 24 h were quantified by RT-qPCR using the 2^−ΔΔCt^ method. No significant changes in mRNA expression were observed for any treatment group compared to control (GPR40: *p* = 0.3288, F = 1.237; PPARγ: *p* = 0.3789, F = 1.114), with data expressed as mean ± SD. GAPDH was used as the housekeeping gene for normalization, after previous tests.

In the Western blot analysis (Figure 3C), a significant reduction in GPR40 protein expression was detected only in the 50 μM 8:2 FTOH group compared with the control group (*p* = 0.0406). This aligns with previous studies reporting interactions between 8:2 FTOH and GPR40 in pancreatic β-cells [13,14,33]. For PPARγ protein expression, no significant differences were observed among any of the treatment groups (*p* = 0.6821, F = 0.5831).

### 3.4. Protein–Protein Interaction (PPI) in Mice by Proteomic Analysis

Proteomic analysis of mouse pancreatic tissue identified 265 total proteins, with 19 upregulated proteins (*p* > 0.95) and 17 downregulated proteins (*p* < 0.05) in the 50 mg/kg 8:2 FTOH group relative to control (Table S2). No unique proteins were identified in any group.

Up-regulated proteins included Chymotrypsinogen B (Q9CR35), Glutathione S-transferase family (P15626, Q8R5I6, Q80W21 and P10649), Hemoglobin subunits (P01942, P02088, P02089 and P02104), and Pancreatic triacylglycerol lipase (Q6P8U6). Downregulated proteins included Alpha-amylase 1 (P00687), several Histone family proteins (Q64475, Q6ZWY9, P10853, Q64478, Q8CGP2, Q8CGP1, P10854, Q64525, Q8CGP0), Colipase and Endoplasmic reticulum chaperone BiP (Q9CQC2 and P20029) (Table S2).

**Figure 4:**
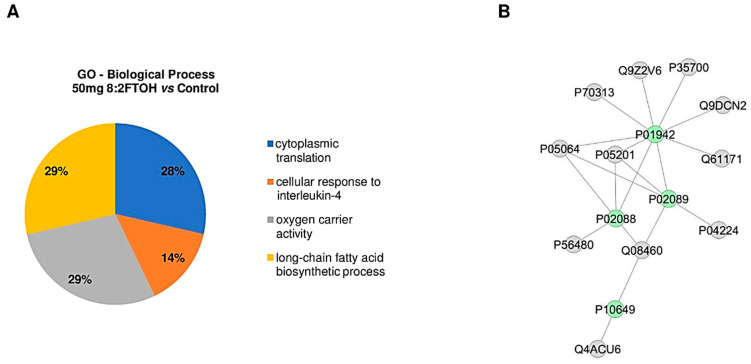
Proteins identified by proteomic analysis. (**A**) Functional distribution of proteins identified with differential expression in the pancreas of male mice treated with 50 mg/kg 8:2 FTOH or not (control group). Categories of proteins based on GO annotation “Biological Process”. Significant terms (Kappa = 0.04) and distribution according to the percentage of number of genes. Protein access number was provided by UNIPROT. Gene ontology was evaluated according to ClueGo^®^ plugins of Cytoscape^®^ software 3.7.1. (**B**) Network generated by ClusterMarker^®^ for the comparison of 50 mg/kg 8:2 FTOH group vs. control group. The color of the node indicates the differential expression of the protein with its access code, obtained from UniProt protein database (http://www.uniprot.org/). Light green nodes indicate upregulated proteins in 50 mg/kg 8:2 FTOH group in comparison to control. Gray nodes represent interaction proteins that are offered by CYTOSCAPE^®^, which were not identified in the present study. *n* = 6 per group.

Functional classification of biological processes revealed that the pathways most affected by 50 mg/kg 8:2 FTOH were the long-chain fatty acid biosynthetic process (29%), oxygen carrier activity (29%) and cytoplasmic translation (28%) (Figure 4A). ClusterMarker identified interactions with protein NADH-cytochrome b5 reductase 3 (Q9DCN2), Histone deacetylase 5 (Q9ZZV6), ATP synthase subunit beta, mitochondria (P56480), and Peroxiredoxin 1 (P35700) and Peroxiredoxin 2 (Q61171) and proteins up-regulated with Hemoglobin subunit alpha (P01942), Hemoglobin subunit beta-2 (P02089), Hemoglobin subunit beta-1 (P02088) and Glutathione S-transferase Mu 1 (P10649) (Figure 4B).

**Figure 5:**
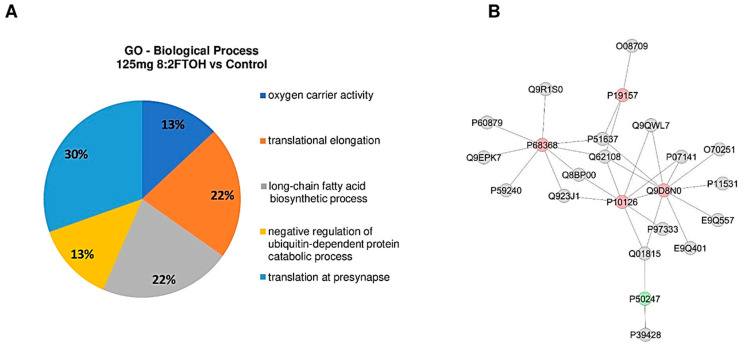
Proteins identified by proteomic analysis. (**A**) Functional distribution of proteins identified with differential expression in the pancreas of male mice treated with 125 mg/kg 8:2 FTOH or not (control group). Categories of proteins based on GO annotation “Biological Process”. Significant terms (Kappa = 0.04) and distribution according to the percentage of number of genes. Protein access number was provided by UNIPROT. Gene ontology was evaluated according to ClueGo^®^ plugins of Cytoscape^®^ software 3.7.1. (**B**) Network generated by ClusterMarker^®^ for the comparison of 125 mg/kg 8:2 FTOH group vs. control group. The color of the node indicates the differential expression of the protein with its access code, obtained from UniProt protein database (http://www.uniprot.org/). Light green nodes indicate upregulated proteins and light red nodes indicate downregulated proteins, respectively, in 125 mg/kg 8:2 FTOH group in comparison to control. Gray nodes represent interaction proteins that are offered by CYTOSCAPE^®^, which were not identified in the present study. *n* = 6 per group.

Comparison of the 125 mg/kg 8:2 FTOH group with control identified 422 total proteins, including 35 upregulated and 54 downregulated proteins (Table S3). ClueGo analysis revealed major affected processes: translation at presynapse (30%), translation elongation (22%) and long-chain fatty acid biosynthetic process (22%) (Figure 5A). ClusterMarker identified interacting proteins as Voltage-dependent L-type calcium channel subunit alpha-1C (Q01815), Ryanodine receptor 2 (E9Q401), Caveolin (P51637), and IQ calmodulin-binding motif-containing protein 1 (Q8BP00), as well as downregulated proteins Tubulin alpha-4A chain (P68368), Glutathione S-transferase P1 (P19157), Elongation factor 1-alpha 1 (P10126), and Elongation factor 1-gamma (Q9D8N0) and up-regulated protein Adenosylhomocysteinase (P50247) (Figure 5B).

**Figure 6:**
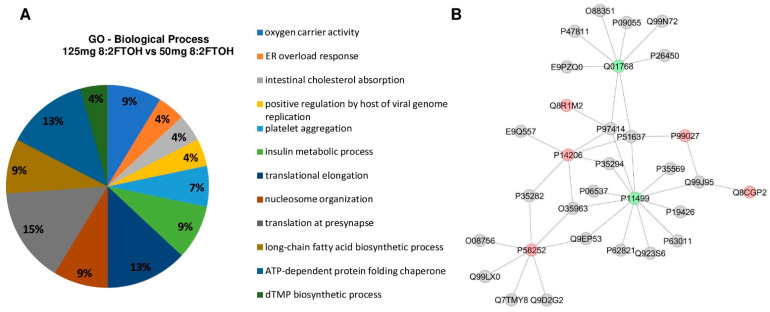
Proteins identified by proteomic analysis. (**A**) Functional distribution of proteins identified with differential expression in the pancreas of male mice treated with 125 mg/kg 8:2 FTOH and 50 mg/kg 8:2 FTOH. Categories of proteins based on GO annotation “Biological Process”. Significant terms (Kappa = 0.04) and distribution according to the percentage of number of genes. Protein access number was provided by UNIPROT. Gene ontology was evaluated according to ClueGo^®^ plugins of Cytoscape^®^ software 3.7.1. (**B**) Network generated by ClusterMarker^®^ for the comparison of 125 mg/kg 8:2 FTOH group vs. 50 mg/kg 8:2 FTOH group. The color of the node indicates the differential expression of the protein with its access code, obtained from UniProt protein database (http://www.uniprot.org/). Light green nodes indicate upregulated proteins and light red nodes indicate downregulated proteins, respectively, in 125 mg/kg 8:2 FTOH group in comparison to 50 mg/kg 8:2 FTOH group. Gray nodes represent interaction proteins that are offered by CYTOSCAPE^®^, which were not identified in the present study. *n* = 6 per group.

When comparing 125 mg/kg vs. 50 mg/kg 8:2 FTOH, 262 total proteins were detected with 14 upregulated and 83 downregulated (Table S4). The most affected processes in the high-dose group were Translation at presynapse (15%), Translation elongation (13%) and ATP-dependent protein folding chaperone (13%) (Figure 6A). ClusterMarker identified proteins interacting with Potassium voltage-gated channel subfamily KQT member 1 (P97414), Caveolin-3 (P51637), Ras-related protein Rab-19 (P35294) and Insulin receptor substrate 1 (P35569) were identified, besides up-regulated proteins were identified in this interactions with Nucleoside diphosphate kinase B (Q01768) and Heat shock protein HSP 90-beta (P11499), as well as downregulated proteins Histone H2A.J (Q8R1M2), Small ribosomal subunit protein uS2 (P14206), Large ribosomal subunit protein P2 (P99027), Histone H2B type 1-P (Q8CGP2) and Elongation factor 2 (P58252) (Figure 6B).

## 4. Discussion

The results of F in urine and plasma, body weight, plasma PFOA, and plasma glucose after 8:2 FTOH exposure will be described in another publication [29]. Briefly, no differences in body weight or plasma glucose were identified among treatment groups. After 8:2 FTOH treatment, its metabolites, PFOA in plasma and F in plasma and urine, were significantly increased in mice compared to the control. Furthermore, it demonstrated the reduction of 8:2 FTOH to fluorinated metabolites in tissues and its excretion by kidneys. These findings demonstrate the reduction of 8:2 FTOH into F in tissues and its renal excretion, which is particularly relevant since the effects of PFAS and their metabolites, including F, on insulin secretion remain poorly understood [13,14,36,37].

A limitation of the present study is the absence of comprehensive metabolic assessments, such as glucose tolerance and insulin sensitivity tests, which would provide further evidence on the functional consequences of 8:2 FTOH exposure in vivo. Our findings do not rule out the possibility of subtle metabolic disturbances that may not be detected by baseline measurements alone.

Despite the growing interest in PFAS toxicity, few studies have investigated PFAS and F at doses simulating human exposure, as most reports use high, overtly toxic concentrations [4,17,36]. Therefore, the present work evaluated physiologically relevant concentrations of F (1 and 5 μM F, as NaF) [32] and non-cytotoxic concentrations of 8:2 FTOH (10 and 50 μM; determined by MTT assay) in βTC-6 cells. LDH assays confirmed the absence of cytotoxicity (Figure 1B), indicating no membrane damage under these conditions.

It should be noted that the U.S. Environmental Protection Agency (EPA) in 2022 recommended lifetime health limits of 70 ng/L (ppt) for combined PFOA and PFOS, but concentrations may be much higher near facilities that manufacture fluoropolymers [38]. In 2022, the EPA updated the interim limit of PFOS to 0.02 ppt and PFOA to 0.004 ppt, which is thousands of times higher than the previous concentration (70 ppt), suggesting that PFOA/PFOS might be more problematic than initially believed.

Regarding insulin levels in both animals’ plasma (*p* = 0.1691, Figure 2C) and β-cells, no significant differences were found (*p* = 0.5012; Figure 2A). This may reflect the short exposure period (24 h) in vitro or biological sex effects in vivo, as sex hormones, adiposity, tissue cellularity, dietary fat, and genetic background can markedly influence insulin sensitivity and secretion [39,40]. Thus, sex-specific responses should be considered in future PFAS–fluoride interaction studies.

Real-time Ca^2+^ intracellular levels analysis in βTC-6 cells showed that the positive control (ionomycin) elicited the expected strong Ca^2+^ response, confirming assay functionality (Figure 2B). In contrast, the negative control (0.04% DMSO) displayed only basal Ca^2+^ levels (*p* < 0.0001). Among the treatments, 5 µM F significantly increased [Ca^2+^]_i_ (*p* < 0.01, Figure 2B), likely due to fluoride’s known affinity for Ca^2+^ and its ability to form stable ionic complexes. This suggests that F may modulate Ca^2+^ homeostasis in pancreatic cells, an essential step in insulin secretion, since [Ca^2+^]_i_ is the primary trigger for insulin granule exocytosis in pancreatic β-cells.

Interestingly, 10 µM 8:2 FTOH significantly elevated [Ca^2+^]_i_ (*p* < 0.05), whereas 50 µM 8:2 FTOH reduced it, indicating a potential biphasic (hormetic) response: lower concentrations may enhance Ca^2+^ signaling, while higher doses impair Ca^2+^ uptake, possibly by inhibiting the Ca^2+^ channels. The lowest F dose (1 µM) produced no significant difference, consistent with a threshold-dependent effect. These finding confirm the safety of controlled water fluoridation, since 1 µM is the plasma F concentration expected to be found in people drinking water containing ~1 mg F/L [41]. This biphasic pattern aligns with evidence that low-dose exposures of F and PFAS may produce subtle yet meaningful effects, even though ~99% of ingested fluoride is deposited in hard tissues [41]. Thus, its influence on soft tissues, particularly pancreatic Ca^2+^ metabolism, remains insufficiently explored. Similar trends have been reported for low-dose PFAS exposures, emphasizing the need to investigate their combined effects on Ca^2+^ homeostasis and insulin regulation.

Previous studies have shown that PFOA can alter insulin secretion through GPR40-mediated signaling [13,36,42]. In our study, although RT-qPCR showed no significant changes in PPARγ and GPR40 gene expression (Figure 3A,B), Western blotting revealed significant downregulation of GPR40 protein at the high dose (50 µM) of 8:2 FTOH (*p* < 0.05, Figure 3C). The absence of changes at the mRNA level suggests post-transcriptional or post-translational regulatory mechanisms. Since GPR40 amplifies insulin secretion [13,39], these findings indicate that high-dose 8:2 FTOH may reduce β-cell sensitivity to glucose through non-transcriptional mechanisms. The lack of effects on PPARγ, a key transcription factor regulating lipid and glucose metabolism [13,43], further supports the idea that rapid protein-level changes may play a central role in FTOH-mediated β-cell alterations. Therefore, changes in [Ca^2+^]_i_ do not necessarily result in measurable alterations in insulin release. Moreover, adaptive cellular repair and compensatory mechanisms may have preserved β-cell function, maintaining the integrity of the insulin exocytosis machinery despite alterations in calcium homeostasis. This interpretation is supported by the reduced GPR40 protein expression observed at 50 µM 8:2 FTOH, indicating that molecular changes can occur in the absence of overt functional impairment. Together, these findings suggest that exposure to 5 µM F and to 10 and 50 µM 8:2 FTOH modulates β-cell calcium homeostasis, while insulin exocytosis remains preserved under the conditions evaluated. Thus, changes in intracellular calcium concentration may represent an early cellular response to environmental contaminants and constitute a more sensitive marker of β-cell dysfunction than insulin secretion itself. Future studies are needed to understand the direct relationship between the specific role of the GPR40 receptor affected by 8:2 FTOH and its response in calcium modulation in pancreatic β cells. In addition, future studies should investigate whether these effects are mediated by extracellular calcium influx, intracellular calcium release, or a combination of both mechanisms. Additional dose–response and temporal kinetic experiments, along with the use of specific inhibitors of calcium channels and signaling pathways, will be important to better characterize the molecular mechanisms underlying the 8:2 FTOH-induced calcium response.

Beale, Sinclair, Shah, Paten, Kumar, Long, Vardy and Jones [43] emphasized in their omics review the need for deeper proteomic studies addressing metabolic dysfunction induced by PFAS exposure. Accordingly, we performed an in vivo proteomic analysis of pancreas from male mice exposed to 50 or 125 mg/kg 8:2 FTOH for 90 days. For the 50 mg/kg comparison versus control, no fold-changes above the commonly accepted 2x threshold were identified [21]. Nonetheless, upregulated proteins involved in lipid metabolism and digestion were identified—such as Pancreatic triacylglycerol lipase (Q6P8U6), Chymotrypsin-like elastase family member 3B (Q9CQ52) and Pancreatic lipase-related protein 2 (P17892) (Table S2)—as described in other studies that showed metabolic changes in lipid metabolism as an effect of PFAS exposure [44,45]. Additionally, proteins involved in detoxification (Glutathione S-transferase Mu 1, 2, 4, 7 (GSTM1, GSTM2, GSTM4, GSTM7)) and cellular energy transport (ADP/ATP translocase 2; P51881) were also upregulated (Table S2). Downregulated proteins included digestive enzymes (Alpha-amylase 1; P00687), Trypsin-4, 5 (Q9R0T7 and Q9QUK9), and several histone family members (Q64475, Q6ZWY9, P10853, Q64478, Q8CGP2, Q8CGP1, P10854, Q64525, Q8CGP0), suggesting possible epigenetic effects, consistent with findings from Trevizol et al. [21]. It was also possible to observe the biological process most affected by the dose of 50 mg/kg 8:2 FTOH being the long-chain fatty acid biosynthetic process (29%) (Figure 4A), and in the PPI network, interacting proteins were involved in the defense of oxidative stress (ROS) (Peroxiredoxin-1 and 2), as well as in the activity of cellular metabolism (NADH-cytochrome b5 reductase 3 and ATP synthase subunit beta, mitochondria) (Figure 4B).

The 125 mg/kg dose of 8:2 FTOH showed upregulated proteins involved in protein quality control, including chaperones (Heat shock protein HSP 90-beta, P11499; Protein disulfide-isomerase A6, Q922R8; and Calreticulin, P14211) (Table S3), indicating potential endoplasmic reticulum (ER) stress, similar to findings in rats chronically exposed to F [46]. Increased expression of ribosomal proteins (P97351, P63325, P62908, P62830, P47962, P62082, and P14131) suggests enhanced protein synthesis or compensation for post-translational modifications. Proteins involved in energy metabolism (Adenosylhomocysteinase, P50247; Nucleoside diphosphate kinases A/B, P15532, Q01768) were also elevated, consistent with increased bioenergetic demand (Table S3).

Conversely, digestive and lipid-related enzymes—including pancreatic alpha-amylase (P00688), pancreatic triacylglycerol lipase (Q6P8U6), and trypsin-4/5 (Q9R0T7, Q9QUK9)—were downregulated by 3-fold, consistent with PFAS-induced metabolic disturbances [42,44,45]. A strong downregulation of multiple histone isoforms (Q64475, Q6ZWY9, P10853, Q64478, Q8CGP1, P10854, Q8CGP2, Q64525, Q64524, Q8CGP0, Q6GSS7, Q64523, Q8BFU2) (Table S3) suggests substantial epigenetic modulation, paralleling findings in NOD mice exposed to low F doses (10 mg/L) [21].

The comparison of 125 mg/kg vs. 50 mg/kg revealed a notable ~6-fold decrease in insulin protein (P01324) (Table S4), highlighting the sensitivity of pancreatic insulin stores to PFAS exposure. The PPI network (Figure 6B) included proteins involved in Ca^2+^ handling and insulin signaling, such as phosphatidylinositol 3-kinase regulatory subunit alpha (PIK3R1) (P26450), Potassium voltage-gated channel subfamily KQT member 1 (KCNQ1) (P97414), Ryanodine receptor 1 (E9PZQ0), and Insulin receptor substrate 1 (IRS-1) (P35569), indicating that high-dose 8:2 FTOH may alter both Ca^2+^ metabolism and insulin production. These findings agree with previous studies [12,13,16].

Based on the exploratory proteomic profiles generated in this study, future research should focus on targeted mechanistic validation of the molecular pathways potentially affected by 8:2 FTOH exposure, particularly those related to oxidative stress, endoplasmic reticulum stress, lipid metabolism, mitochondrial function, calcium homeostasis, and GPR40 signaling, using complementary approaches such as Western blotting, RT-qPCR, ELISA, or targeted proteomics. Additionally, future investigations should expand these analyses to female models, given that sex-dependent differences may influence glucose homeostasis, insulin signaling, and responses to environmental contaminants. In this regard, complementary proteomic and plasma insulin analyses in female mice are currently under investigation in our laboratory and will be reported in a future study, contributing to a better understanding of potential sex-specific molecular responses to 8:2 FTOH exposure. Moreover, future investigations employing additional doses and time points, as well as pharmacological inhibitors of calcium signaling pathways, are required to clarify whether the response originates from extracellular calcium influx or intracellular calcium release.

Interestingly, GPR40 protein was not identified in the pancreas by proteomic analysis, regardless of treatment. This may reflect dose limitations, tissue heterogeneity, or the presence of GPR40 predominantly in β-cells rather than whole pancreatic tissue, underscoring the importance of cell-specific proteomic studies to better understand PFAS-induced pancreatic dysfunction. 

## 5. Conclusions

In conclusion, exposure to 8:2 FTOH modulates Ca^2+^ homeostasis in pancreatic β-cells in a dose-dependent, biphasic manner, resembling the response observed for F, and at higher concentrations leads to downregulation of the GPR40 protein. In vivo proteomics analysis further revealed alterations in proteins and interaction networks associated with pathways involved in lipid metabolism, endoplasmic reticulum stress, and Ca^2+^ signaling. Together, these findings suggest that PFAS and F, at the highest doses, can influence Ca^2+^ homeostasis in pancreatic tissue and may affect insulin secretion through complex mechanisms that warrant further investigation. On the other hand, the low doses did not elicit substantial alterations, which is in line with the hormesis effect of F [47]. Our findings provide additional support for the safety of controlled PFAS exposure.

## Data Availability

The raw data supporting the conclusions of this article will be made available by the authors on request.

## Supplementary Materials

The following supporting information can be downloaded at https://www.mdpi.com/article/10.3390/metabo16070470/s1: Table S1: Primer sequences; Table S2: Protein identified by LC-MS/MS 50 mg/kg 8:2 FTOH vs. Control; Table S3: Protein identified by LC-MS/MS 125 mg/kg 8:2 FTOH vs. Control; Table S4: Protein identified by LC-MS/MS 125 mg/kg 8:2 FTOH vs. 50 mg/kg 8:2 FTOH.

## Abbreviations

The following abbreviations are used in this manuscript:

8:2 FTOH	8:2 Fluorotelomer alcohol
ADP	Adenosine diphosphate
ATP	Adenosine triphosphate
βTC-6	Mouse pancreatic β-cell line
Ca^2+^	Calcium ion
DMSO	Dimethyl sulfoxide
EPA	Environmental Protection Agency
ER	Endoplasmic reticulum
F	Fluoride
GPR40	G protein-coupled receptor 40
GSTM	Glutathione S-transferase Mu
HSP90	Heat shock protein 90
IRS-1	Insulin receptor substrate 1
KCNQ1	Potassium voltage-gated channel subfamily KQT member 1
LDH	Lactate dehydrogenase
MTT	3-(4,5-Dimethylthiazol-2-yl)-2,5-diphenyltetrazolium bromide
NaF	Sodium fluoride
NADH	Nicotinamide adenine dinucleotide (reduced form)
PFAS	Per- and polyfluoroalkyl substances
PFOS	Perfluorooctane sulfonate
PFOA	Perfluorooctanoic acid
PIK3R1	Phosphatidylinositol 3-kinase regulatory subunit alpha
PPI	Protein–protein interaction
PPARγ	Peroxisome proliferator-activated receptor gamma
ppt	Parts per trillion
qPCR	Quantitative polymerase chain reaction
ROS	Reactive oxygen species
RT-qPCR	Reverse transcription quantitative polymerase chain reaction
