# Supplementary material for: Effects of Fluoride and 8:2 FTOH on β-Cell Calcium Signaling and Insulin Homeostasis: An Exploratory Study
Source: Metabolites. 2026 Jul 4;16(7):470. doi: 10.3390/metabo16070470 (PMC13413545; doi:10.3390/metabo16070470)
Supplement: Supplementary file 1 [file metabolites-16-00470-s001.zip › metabolites-4375232-supplementary.pdf]

Supplementary Table

Table S1 Primer sequences

Gene	GenBank ID#	5' Primer	3' Primer
FFAR1	NM_194057.3	CTGGGCATCAACATACCCGT	AGCAGAAGGCAGTGATGACC
PPAR-γ	NM_001127330.3	CTGTGAGACCAACAGCCTGAC	TCAGTGGTTCACCGCTTCTTT
GAPDH	NM_001289726.2	GCAAAGTGGAGATTGTTGCCAT	CCTTGACTGTGCCGTTGAATTT

Table S2 Protein identified by LC-MS/MS 50 mg/kg 8:2 FTOH vs. Control

[a]Access Number	Protein name	PLGS score	[b]*Ratio* 50 mg/kg 8:2 FTOH Control
P02089	Hemoglobin subunit beta-2	604	1.97
P01942	Hemoglobin subunit alpha	253	1.75
P02104	Hemoglobin subunit epsilon-Y2	604	1.58
P02088	Hemoglobin subunit beta-1	798	1.52
P62843	Small ribosomal subunit protein uS19	2735	1.43
P51881	ADP/ATP translocase 2	221	1.40
P15626	Glutathione S-transferase Mu 2	612	1.38
Q8R5I6	Glutathione S-transferase Mu 4	408	1.34
Q80W21	Glutathione S-transferase Mu 7	546	1.28
P17892	Pancreatic lipase-related protein 2	55	1.27
Q8VCK7	Syncollin	300	1.26
P10649	Glutathione S-transferase Mu 1	857	1.25
P97351	Small ribosomal subunit protein eS1	358	1.23
P00683	Ribonuclease pancreatic	628	1.22
P99027	Large ribosomal subunit protein P2	6414	1.19
Q9CR35	Chymotrypsinogen B	3631	1.12
Q9CQ52	Chymotrypsin-like elastase family member 3B	9301	1.06
Q6P8U6	Pancreatic triacylglycerol lipase	498	1.05
P00687	Alpha-amylase 1	735	0.98
P20029	Endoplasmic reticulum chaperone BiP	543	0.93
P10853	Histone H2B type 1-F/J/L	2075	0.92
Q64478	Histone H2B type 1-H	2075	0.92
Q8CGP2	Histone H2B type 1-P	2075	0.92
Q64475	Histone H2B type 1-B	2075	0.91
Q6ZWY9	Histone H2B type 1-C/E/G	2075	0.91
Q9CQC2	Colipase	325	0.91
Q8CGP1	Histone H2B type 1-K	2075	0.91
P10854	Histone H2B type 1-M	2075	0.91
Q64525	Histone H2B type 2-B	2075	0.91

Q9R0T7	Trypsin-4	847	0.91
Q9QUK9	Trypsin-5	882	0.91
Q8CGP0	Histone H2B type 3-B	2075	0.88
Q9D2U9	H2B.U histone 2	2075	0.88
Q64524	Histone H2B type 2-E	2075	0.88
P25444	Small ribosomal subunit protein uS5	789	0.87

[a] Identification is based on proteins ID from UniProt protein databases. Reviewed only (http://www.uniprot.org/).

[b] Protein with expression significantly altered are organized according to the ratio

Table S3 Protein identified by LC-MS/MS 125 mg/kg 8:2 FTOH vs. Control

[a]Access Number	Protein name	PLGS score	[b]*Ratio* 125 mg/kg 8:2 FTOH Control
P50247	Adenosylhomocysteinase	274	1.92
P51881	ADP/ATP translocase 2	221	1.63
Q9CQ52	Chymotrypsin-like elastase family member 3B	9301	1.63
P10649	Glutathione S-transferase Mu 1	857	1.57
P15626	Glutathione S-transferase Mu 2	612	1.49
Q80W21	Glutathione S-transferase Mu 7	546	1.43
P11499	Heat shock protein HSP 90-beta	12	1.43
P70696	Histone H2B type 1-A	505	1.36
P51410	Large ribosomal subunit protein uL6	276	1.35
Q9D154	Leukocyte elastase inhibitor A	219	1.34
P43137	Lithostathine-1	393	1.34
P15532	Nucleoside diphosphate kinase A	544	1.31
Q01768	Nucleoside diphosphate kinase B	544	1.30
P97351	Small ribosomal subunit protein eS1	358	1.30
P63325	Small ribosomal subunit protein eS10	226	1.30
P62908	Small ribosomal subunit protein Us3	1854	1.28
Q8R5I6	Glutathione S-transferase Mu 4	408	1.28
P02089	Hemoglobin subunit beta-2	604	1.28
P62830	Large ribosomal subunit protein uL14	483	1.27
P47962	Large ribosomal subunit protein uL18	373	1.27
P17892	Pancreatic lipase-related protein 2	55	1.27
P62270	Small ribosomal subunit protein uS13	439	1.27
Q8VCK7	Syncollin	300	1.27
Q62186	Translocon-associated protein subunit delta	644	1.25
P68368	Tubulin alpha-4A chain	330	1.25
P14211	Calreticulin	302	1.23
Q922R8	Protein disulfide-isomerase A6	408	1.23
Q9D8N0	Elongation factor 1-gamma	143	1.22
Q6ZWU9	Small ribosomal subunit protein eS27	2175	1.22
P62245	Small ribosomal subunit protein uS8	2486	1.21

P63242	Eukaryotic translation initiation factor 5A-1	667	1.20
P19639	Glutathione S-transferase Mu 3	185	1.20
P02088	Hemoglobin subunit beta-1	798	1.19
P62082	Small ribosomal subunit protein eS7	257	1.15
P14131	Small ribosomal subunit protein uS9	973	1.08
O35855	Branched-chain-amino-acid aminotransferase_ mitochondrial	271	0.91
P16858	Glyceraldehyde-3-phosphate dehydrogenase	276	0.90
P15947	Kallikrein-1	139	0.90
P01942	Hemoglobin subunit alpha	253	0.88
P14206	Small ribosomal subunit protein uS2	981	0.88
P60710	Actin_ cytoplasmic 1	1096	0.85
P19001	Keratin_ type I cytoskeletal 19	203	0.84
D3Z6P0	Protein disulfide-isomerase A2	300	0.79
P00687	Alpha-amylase 1	735	0.71
P07146	Anionic trypsin-2	4165	0.71
P56480	ATP synthase subunit beta_ mitochondrial	125	0.71
Q8BFZ3	Beta-actin-like protein 2	455	0.70
Q64285	Bile salt-activated lipase	331	0.68
Q7TPZ8	Carboxypeptidase A1	135	0.62
Q91X79	Chymotrypsin-like elastase family member 1	368	0.62
P05208	Chymotrypsin-like elastase family member 2A	2463	0.58
Q9CR35	Chymotrypsinogen B	3631	0.57
P10126	Elongation factor 1-alpha 1	516	0.57
P62631	Elongation factor 1-alpha 2	140	0.57
P20029	Endoplasmic reticulum chaperone BiP	543	0.57
P19157	Glutathione S-transferase P 1	310	0.56
Q9D2U9	H2B.U histone 2	2075	0.56
C0HKE1	Histone H2A type 1-B	260	0.56
C0HKE2	Histone H2A type 1-C	260	0.56
C0HKE3	Histone H2A type 1-D	260	0.56
C0HKE4	Histone H2A type 1-E	260	0.54
Q8CGP5	Histone H2A type 1-F	260	0.54
C0HKE5	Histone H2A type 1-G	260	0.54
Q8CGP6	Histone H2A type 1-H	260	0.52
C0HKE6	Histone H2A type 1-I	260	0.51
Q8CGP7	Histone H2A type 1-K	260	0.50
C0HKE7	Histone H2A type 1-N	260	0.50
C0HKE8	Histone H2A type 1-O	260	0.43
C0HKE9	Histone H2A type 1-P	260	0.42
Q6GSS7	Histone H2A type 2-A	260	0.41
Q64523	Histone H2A type 2-C	260	0.41
Q8BFU2	Histone H2A type 3	260	0.41
Q8R1M2	Histone H2A.J	260	0.41
Q64475	Histone H2B type 1-B	2075	0.40
Q6ZWY9	Histone H2B type 1-C/E/G	2075	0.40

P10853	Histone H2B type 1-F/J/L	2075	0.40
Q64478	Histone H2B type 1-H	2075	0.40
Q8CGP1	Histone H2B type 1-K	2075	0.40
P10854	Histone H2B type 1-M	2075	0.40
Q8CGP2	Histone H2B type 1-P	2075	0.40
Q64525	Histone H2B type 2-B	2075	0.40
Q64524	Histone H2B type 2-E	2075	0.40
Q8CGP0	Histone H2B type 3-B	2075	0.40
P47955	Large ribosomal subunit protein P1	632	0.40
P00688	Pancreatic alpha-amylase 2a5 O	3221	0.39
Q6P8U6	Pancreatic triacylglycerol lipase	498	0.39
P00683	Ribonuclease pancreatic	628	0.36
Q9R0T7	Trypsin-4	847	0.35
Q9QUK9	Trypsin-5	882	0.32
Q8K0C5	Zymogen granule membrane protein 16	420	0.31

[a] Identification is based on proteins ID from UniProt protein databases. Reviewed only (http://www.uniprot.org/).

[b] Protein with expression significantly altered are organized according to the ratio

Table S4 Protein identified by LC-MS/MS 125 mg/kg 8:2 FTOH vs. 50 mg/kg 8:2 FTOH

[a]Access Number	Protein name	PLGS score	[b]*Ratio* 125 mg/kg 8:2 FTOH 50 mg/kg 8:2 FTOH
P11499	Heat shock protein HSP 90-beta	19	1.43
P51410	Large ribosomal subunit protein uL6	277	1.39
P62245	Small ribosomal subunit protein uS8	1983	1.35
P47962	Large ribosomal subunit protein uL18	230	1.30
P14131	Small ribosomal subunit protein uS9	814	1.25
P15532	Nucleoside diphosphate kinase A	1104	1.22
Q01768	Nucleoside diphosphate kinase B	1097	1.22
P00683	Ribonuclease pancreatic	4643	1.12
P08113	Endoplasmin	460	1.08
Q99PL5	Ribosome-binding protein 1	61	1.16
Q9CR35	Chymotrypsinogen B	16045	1.05
P43137	Lithostathine-1	374	1.19
P62702	Small ribosomal subunit protein eS4	603	1.12
Q922R8	Protein disulfide-isomerase A6	421	1.09
P16627	Heat shock 70 kDa protein 1-like	296	0.91
P63323	Small ribosomal subunit protein eS12 O	743	0.90
P51881	ADP/ATP translocase 2	86	0.90
P68033	Actin_ alpha cardiac muscle 1	676	0.89

O08810	116 kDa U5 small nuclear ribonucleoprotein component	94	0.89
Q8VCR2	17-beta-hydroxysteroid dehydrogenase 13	203	0.88
P62849	Small ribosomal subunit protein eS24	292	0.88
Q8R5I6	Glutathione S-transferase Mu 4	1028	0.88
Q80W21	Glutathione S-transferase Mu 7	1225	0.88
P14206	Small ribosomal subunit protein uS2	2358	0.87
P08249	Malate dehydrogenase_ mitochondrial	261	0.87
P17879	Heat shock 70 kDa protein 1B	195	0.87
P01326	Insulin-2	655	0.86
P07724	Albumin	3279	0.86
P58252	Elongation factor 2	1877	0.86
P10649	Glutathione S-transferase Mu 1	1547	0.86
Q03265	ATP synthase subunit alpha_ mitochondrial	360	0.85
P62806	Histone H4	8196	0.83
P05208	Chymotrypsin-like elastase family member 2A	13805	0.82
P15626	Glutathione S-transferase Mu 2	1397	0.80
P00687	Alpha-amylase 1	8090	0.80
P68040	Small ribosomal subunit protein RACK1 O	1826	0.79
O88986	2-amino-3-ketobutyrate coenzyme A ligase_ mitochondrial	262	0.76
Q5BKQ4	Inactive pancreatic lipase-related protein 1	2106	0.76
D3Z6P0	Protein disulfide-isomerase A2	6600	0.75
P63260	Actin_ cytoplasmic 2	1232	0.74
P60710	Actin_ cytoplasmic 1	1232	0.73
P56480	ATP synthase subunit beta_ mitochondrial	1076	0.72
Q8K0C5	Zymogen granule membrane protein 16	2399	0.71
Q61696	Heat shock 70 kDa protein 1A	195	0.70
P99027	Large ribosomal subunit protein P2	6472	0.69
P24369	Peptidyl-prolyl cis-trans isomerase B	1822	0.59
P02089	Hemoglobin subunit beta-2	8669	0.56
Q9R0T7	Trypsin-4	2942	0.55
Q64478	Histone H2B type 1-H	5673	0.55
Q9QUK9	Trypsin-5	3869	0.54
P10854	Histone H2B type 1-M	5673	0.54
Q64524	Histone H2B type 2-E	4696	0.54
P02104	Hemoglobin subunit epsilon-Y2	1859	0.52
P01942	Hemoglobin subunit alpha	11837	0.51
P02088	Hemoglobin subunit beta-1	14178	0.50
P70696	Histone H2B type 1-A	1491	0.47
P62631	Elongation factor 1-alpha 2	2901	0.46
P10853	Histone H2B type 1-F/J/L	5673	0.45
P20029	Endoplasmic reticulum chaperone BiP	3992	0.45
Q64475	Histone H2B type 1-B	5673	0.45
Q6ZWY9	Histone H2B type 1-C/E/G	5673	0.45
Q8CGP1	Histone H2B type 1-K	5673	0.45
Q64525	Histone H2B type 2-B	5673	0.45

Q8CGP2	Histone H2B type 1-P	5673	0.44
Q9D2U9	H2B.U histone 2	4696	0.44
Q64522	Histone H2A type 2-B	1109	0.44
Q8CGP0	Histone H2B type 3-B	4696	0.44
P10126	Elongation factor 1-alpha 1	10092	0.44
P09103	Protein disulfide-isomerase	8932	0.42
P0C0S6	Histone H2A.Z	1538	0.42
P27661	Histone H2AX	1538	0.42
Q3THW5	Histone H2A.V	1538	0.39
C0HKE4	Histone H2A type 1-E	4427	0.39
C0HKE7	Histone H2A type 1-N	4427	0.39
C0HKE3	Histone H2A type 1-D	4427	0.39
Q8CGP5	Histone H2A type 1-F	4427	0.39
Q8CGP6	Histone H2A type 1-H	4427	0.39
C0HKE6	Histone H2A type 1-I	4427	0.39
Q8CGP7	Histone H2A type 1-K	4427	0.39
Q6GSS7	Histone H2A type 2-A	4427	0.39
Q64523	Histone H2A type 2-C	4427	0.39
Q8BFU2	Histone H2A type 3	4427	0.39
Q8R1M2	Histone H2A.J	4427	0.38
Q9CQC2	Colipase	3553	0.38
C0HKE1	Histone H2A type 1-B	4427	0.38
C0HKE5	Histone H2A type 1-G	4427	0.38
C0HKE8	Histone H2A type 1-O	4427	0.38
P07146	Anionic trypsin-2	13658	0.38
C0HKE2	Histone H2A type 1-C	4427	0.38
C0HKE9	Histone H2A type 1-P	4427	0.36
Q9CQ52	Chymotrypsin-like elastase family member 3B	17459	0.36
Q64285	Bile salt-activated lipase	3522	0.34
P00688	Pancreatic alpha-amylase 2a5	35732	0.33
Q6P8U6	Pancreatic triacylglycerol lipase	10137	0.31
P47955	Large ribosomal subunit protein P1	4074	0.28
Q91X79	Chymotrypsin-like elastase family member 1	15033	0.23
P01324	Insulin	655	0.22

[a] Identification is based on proteins ID from UniProt protein databases. Reviewed only (http://www.uniprot.org/).

[b] Protein with expression significantly altered are organized according to the ratio
